# Dr. Cornelius George Comegys

**Published:** 1896-03

**Authors:** 


Dr. Cornelius George Comegys, of Cincinnati, died at his residence February 10, 1896, aged 79 years. He was a man of conspicuous prominence in his profession, a valued citizen and an active man in the social circle. He was prominent in medical society work, local, state and national, and frequently contributed to medical literature. He worked up to the last and fell ill with grip, which, together with its complications, proved too great for his naturally strong constitution, now necessarily weakened by advancing years. He leaves a name to be envied and an example to be emulated by all young physicians.

